# Entropy Indicators: An Approach for Low-Speed Bearing Diagnosis

**DOI:** 10.3390/s21030849

**Published:** 2021-01-27

**Authors:** Diego Sandoval, Urko Leturiondo, Yolanda Vidal, Francesc Pozo

**Affiliations:** 1 Ikerlan Technology Research Centre, Basque Research and Technology Alliance (BRTA), Pº. J. Mª. Arizmendiarrieta, 2, 20500 Arrasate-Mondragón, Spain; dasandoval@ikerlan.es; 2 Control, Modeling, Identification, and Applications (CoDAlab), Department of Mathematics, Escola d’Enginyeria de Barcelona Est (EEBE), Campus Diagonal-Besòs (CDB), Universitat Politècnica de Catalunya (UPC), Eduard Maristany, 16, 08019 Barcelona, Spain; yolanda.vidal@upc.edu (Y.V.); francesc.pozo@upc.edu (F.P.)

**Keywords:** pitch bearing, condition monitoring, entropy, low-speed bearings, vibration

## Abstract

To increase the competitiveness of wind energy, the maintenance costs of offshore floating and fixed wind turbines need to be reduced. One strategy is the enhancement of the condition monitoring techniques for pitch bearings, because their low operational speed and the high loads applied to them make their monitoring challenging. Vibration analysis has been widely used for monitoring the bearing condition with good results obtained for regular bearings, but with difficulties when the operational speed decreases. Therefore, new techniques are required to enhance the capabilities of vibration analysis for bearings under such operational conditions. This study proposes the use of indicators based on entropy for monitoring a low-speed bearing condition. The indicators used are approximate, dispersion, singular value decomposition, and spectral entropy of the permutation entropy. This approach has been tested with vibration signals acquired in a test rig with bearings under different health conditions. The results show that entropy indicators (EIs) can discriminate with higher-accuracy damaged bearings for low-speed bearings compared with the regular indicators. Furthermore, it is shown that the combination of regular and entropy-based indicators can also contribute to a more reliable diagnosis.

## 1. Introduction

Of all existing sources of renewable energy, wind energy is considered to have by the European Union (EU) the largest potential to replace the fossil energy share in the next 30 years [1]. With an estimated required energy production ranging between 230 and 450 GW in Europe by 2050 [2], and with 60% of the investment in renewable energy projects in Europe focusing only on offshore wind projects [3], with the achievement of lower energy prices is a strategy to promote these projects.

Given that almost one-third of the price of 1 MWh from a wind farm is related to the payment of the operating and maintenance (O&M) fixed cost [4], new strategies are needed to reduce the impact of the mentioned cost and to consequently promote wind energy investments. One strategy is condition-based maintenance (CBM), which can alleviate the O&M fixed cost when using a preventive maintenance approach. For convenience, the definitions from standard EN 13372 [5] and EN 13306 [6] will be used. condition-based maintenance (CBM) is maintenance that is performed as governed by condition monitoring (CM) programs, which are the acquisition and processing of information and data that indicate the state of a part or machine over time. One vital aspect of CM is the diagnosis, which is known as the action taken for fault recognition, fault location, and cause identification [6]. Therefore, the diagnosis is fundamental to the achievement of CM programs.

**Figure 1:**
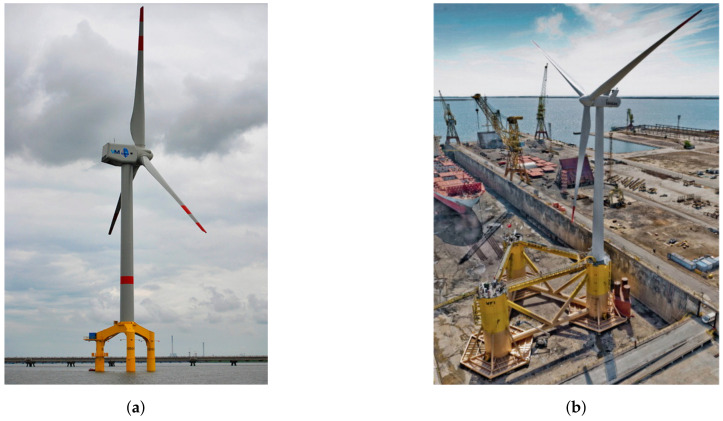
Offshore wind turbines. (**a**) Wind turbine over a fixed foundation [14]. (**b**) Floating offshore wind turbine assembly [15].

For wind turbines (Figure 1), where several electromechanical parts are involved, bearings are critical elements as they work under varying conditions and high loads. For example, 70% of the downtime caused by the gearbox and the generator are based on a bearing malfunction [7]. Thus, the condition monitoring (CM) publications of wind turbine bearings are mainly focused on gearbox and generators [8,9,10]. From the bearings present on wind turbines, the pitch bearings are responsible for the pitch movement of the blade therefore, they are fundamental for energy generation control. While regular bearings have diameters that are of the order of centimeters and rotational speeds that are of the order of several thousand revolutions per minute (rpm), pitch bearings are larger (diameters of the order of meters) and operate at lower rotational speeds (10 rpm and below). As mentioned before, pitch bearings are under severe mechanical stress, with loads and moments of several hundreds of kN and thousands of kNm, respectively, [11,12,13]. Thus, it is particularly challenging to determine condition monitoring (CM) for this type of bearing because of the circumstances previously described.

A common approach for the diagnosis of the bearing is vibration analysis [16,17], as the characteristics in the time and frequency domains of the vibrational response of a bearing change according to its condition [18,19]. The changes mentioned can be noted in the time domain using several techniques. One regular approach found in the literature is the use of signal decomposition algorithms, such as empirical mode decomposition (EMD) [20] and its derivatives ensemble and complementary ensemble EMD [21,22,23], and autoregressive models [24]. Some other algorithms are inspired by EMD, such as adaptive local iterative filtering [25] and fast iterative filtering decomposition [26]. With respect to the frequency domain, the decomposition of the signal by the Fourier transform [27], the Hilbert–Huang transform (based on EMD) [28], and the continuous wavelet transform [29] can be found. In addition, the analysis of the signal by the kurtogram application [30] or spectrogram [31] are used to detect changes in the frequency characteristics. In general, the decomposition of the vibration signals for detecting and diagnosing low-speed bearing damage are not comparable to the results obtained by their application to regular bearings [32]. The main reason for this is the low energy release from the damaged bearing because of the low speed, which can be masked by background noise [33].

A different procedure for fault detection is the calculation of indicators. The kurtosis, covariance, root mean square (RMS), and skewness are indicators that are widely used in the literature [34,35]. In addition, other indicators have been developed in the time domain, such as factors [36,37,38], and Hjorth parameters [39,40,41]. In the frequency domain, several statistical indicators have their counterparts from the time domain, which are helpful for frequency analysis [42]. Using indicators and the aforementioned techniques, the diagnosis of bearings is usually achieved [40,43,44]. However, these indicators also face the problem of low-speed bearings and their low release of energy to detect possible damage. Therefore, new types of indicators are needed to obtain more accurate information from the vibration signals of low-speed bearings.

An interesting group of indicators is especially appealing for bearing diagnosis, namely entropy-based indicators. These types of indicators have been used with promising results in different fields, such as biomedicine [45,46,47,48], economics [49,50], electronics [51], and computer science [52]. The use of entropy-based indicators in vibration analysis can be found in the literature, such as the work by Fei et al. [53], where entropy-based distances are applied for the analysis of vibration signals of rolling element bearing faults. In that work, several spectrum-based indicators are extracted from simulated faulty vibration signals for diagnosis. The research work of Gu et al. [54] uses the minimum average envelope entropy with the Teager energy operator method to diagnose incipient faults on bearings. Zhang et al. [55] decomposed vibration signals from a bearing at speeds ranging from 1720 to 1797 rpm into intrinsic mode functions, to later calculate several multiscale entropy indicators, and to finally diagnose the bearings. Qin et al. [56] used EMD and the energy entropy to select the intrinsic mode functions (IMF) component for feature extraction and subsequently bearing diagnosis. The work by Wang et al. [57] combined the wavelet packet to decompose a vibration signal, select a component according to the entropy indicator criteria, and later extract information of the selected component by using classic indicators (CIs). A similar process was followed by Vakharia et al. [58], where a multiscale entropy-based indicator was used. In general, entropy-based methods are employed for the selection of components from signal decomposition algorithms to avoid noise filtering. Overall, the use of these techniques is applied mainly for general-purpose bearings, with rotational speeds of several thousand rpm.

A small number of articles in the literature present the use of entropy-based indicators in combination with several techniques previously mentioned, such as Huo et al. [59], who used an adaptive multiscale weighted entropy and a support vector machine for the pattern recognition of damaged bearings. Yang et al. [60] worked with a multiscale symbolic dynamic entropy indicator in combination with a support tensor machine-based binary tree for health condition identification. Another example is the work by Fu et al. [61], who used a composite multiscale fine-sorted dispersion entropy, and principal component analysis for the fault diagnosis of bearings. This number decreases for articles referred to as low-speed bearing diagnosis. In this field, Caesarendra et al. [62,63] presents the application of nonlinear methods of feature extraction, which includes one entropy-based method to a bearing at 1 rpm. An et al. [64] utilized variational mode decomposition, which was later combined with a permutation entropy for fault diagnosis of a bearing at 260 rpm.

Although multiple articles use one entropy indicator for fault diagnosis, according to the published literature, the use of several entropy-based indicators for the classification of low-speed bearings is not capable of achieving damage localization. Consequently, the contribution of this study is the research on the feasibility of a set of entropy-based indicators for the classification and diagnosis of low-speed bearings with no previous signal decomposition or filtering. An advantage to highlight in the present work is the absence of signal preprocessing before the calculation of the indicators. From this perspective, a limited number of articles can claim this advantage, such as the work from Glowacz et al. [65], where the calculation of indicators based on the differences between frequency spectra of the vibration signals from different damage scenarios is proposed. From the use of several entropy-based indicators, the information extracted is compared to a set of classical indicators. To analyze how entropy-based and CIs can complement each other, the combination of both types of indicators is studied. As the RF classifier provides excellent performance in pattern recognition [66,67,68,69,70], this study attempts to utilize the RF classifier to construct a classification model for fault recognition of low-speed bearings.

The overall structure of the paper has been divided into six sections, including this introductory section. Section 2 describes the experimental setup, followed by the description of the theory behind the entropy and statistical indicators. The methodology is explained in Section 4, and the results and their discussion are presented in Section 5. Finally, conclusions are given in Section 6.

## 2. Experimental Setup

**Figure 2:**
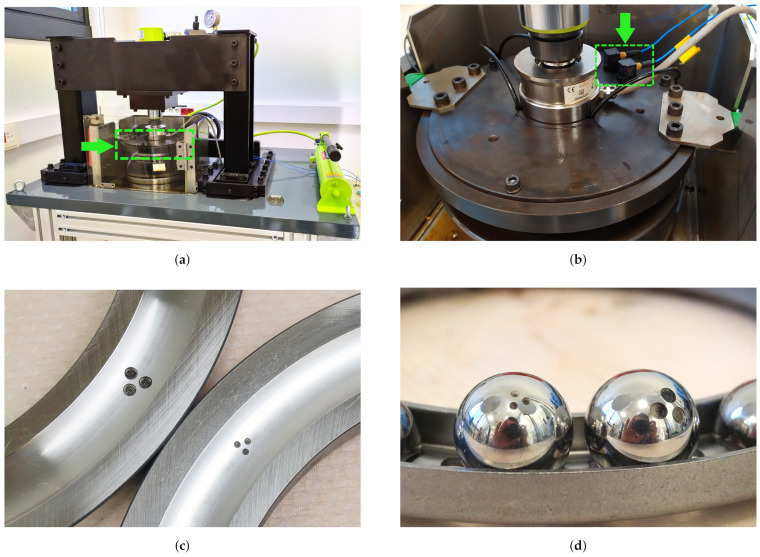
Test rig for low-speed bearings: (**a**) General view of the test rig [74]. The position of the upper support disc is highlighted with a green arrow. (**b**) Detail of the surface of the upper support disc. The position of the sensor is highlighted with a green arrow. (**c**) Damage seeded on shaft washers. (**d**) Damage seeded on ball bearings.

Whereas open data sets are available for regular bearings in different repositories [71,72,73], low-speed bearing data are scarce and usually proprietary. In this work, to evaluate the proposed strategy, a dataset of vibration signals from low-speed bearings was obtained via an experimental test rig using thrust ball bearings [74] (Figure 2a). Thus, the specific bearing operating conditions were established according to the literature [22,75,76]: (i) The bearings rotated at a very low speed (between 5 and 10 rpm), and (ii) the test rig applied a high force (50 kN) to the bearing.

The vibration signals were acquired via a 356A16 ICP acceleration sensor from PCB Piezotronics^®^ (highlighted by a green arrow in Figure 2b), which was placed using a wax adhesive on the surface of the upper support disc (highlighted by a green arrow in Figure 2a). A cDAQ 9174 data acquisition system from the National Instruments^®^ was used to acquire the raw vibration signal from the sensor at a sampling rate of 10.24 kHZ.

The described experimental setup was used to obtain a dataset with healthy and damaged scenarios at different constant rotational speeds of 5, 8, and 10 rpm. The damaged scenarios include different locations and sizes thus, five FAG 51226 thrust ball bearings were used. With respect to location, damage to the shaft washer and ball bearing was introduced. Moreover, small and large damage sizes were considered for both locations. The damage used in each scenario was seeded as the shape of three circles having the same diameter and 0.3 mm deep by electro-discharge machining on the surface of the element, with special considerations for the curvature in the raceway of the shaft washer (Figure 2c) and ball bearing (Figure 2d). The circles were equally spaced by 1 mm from each other, and different diameters were used according to the damage size: 1.5 mm for small damage, and 3 mm for large damage.

In summary, the data set included time signals for different speeds (5, 8, and 10 rpm) in each of the five scenarios, which are denoted as follows: healthy scenario (HS), small raceway shaft washer damage scenario (RS), large raceway shaft washer damage scenario (RL), small ball bearing damage scenario (BS), and ball bearing damage scenario (BL).

## 3. Theoretical Background: Classic and Entropy Indicators

This section presents a brief review of the most common indicators. First, a selection of indicators found in the literature is summarized in Section 3.1. Secondly, an explanation of the proposed indicators based on entropy is presented in Section 3.2.

### 3.1. Classic Indicators

The reviews in the literature summarize that to obtain information on the state of a low-speed bearing, several indicators can be calculated [7,16,17,77,78,79,80,81,82,83]. From all indicators found in the literature, a specific group is regularly used, which are referred to the remainder of this article as CIs. For the sake of clarity, a sampled vibration signal defined as $x=\{x_{1},x_{2},\ldots ,x_{N}\},\;N\in N$ is used for the mathematical description of CIs.

Many of the selected CIs are computed in the time domain. From all indicators, the most widely known and used is the RMS, which is calculated as:

(1) $$\begin{array}{c} \mathrm{RMS}=\sqrt{\frac{1}{N}\sum_{i=1}^{N}x_{i}^{2}}. \end{array}$$

In several cases, the value of RMS increases as the faults in a bearing are developed [34,84]. As this statement is not true for all types of bearings and faults, researchers are forced to find more reliable indicators. Complementary to RMS, the upper bound $h_{u}$ and lower bound $h_{l}$ of the histograms of the vibration signal are also indicators that may be sensitive to changes in the state of bearings [79,80,85]. These indicators are calculated as follows:

(2) $$\begin{array}{cc} h_{u} & =\max_{i=1,\ldots ,N}\left(x_{i}\right)+\frac{\Delta }{2}, \end{array}$$

(3) $$\begin{array}{cc} h_{l} & =\max_{i=1,\ldots ,N}\left(x_{i}\right)-\frac{\Delta }{2}, \end{array}$$

where

$$\begin{array}{cc} \Delta & =\frac{\max_{i=1,\ldots ,N}\left(x_{i}\right)-\min_{i=1,\ldots ,N}\left(x_{i}\right)}{N-1}. \end{array}$$

Other indicators in the literature are specifically designed (but not limited) for bearings, such as shape factor (SF), crest factor (CF), impulse factor (IF), and margin factor (MF) [37,86]. By definition, SF is related to the shape of the signal independent of its time extension. The calculation is performed as follows:

(4) $$\begin{array}{c} \mathrm{SF}=\frac{\sqrt{\frac{1}{N}\sum_{i=1}^{N}x_{i}^{2}}}{\frac{1}{N}\sum_{i=1}^{N}\left|x_{i}\right|}. \end{array}$$

In contrast to SF, CF and IF measure the impulsiveness of the signal from the bearing. Therefore, both indicators are specially used for spiky signals. Although the mathematical formulation for both indicators is similar, the main difference is the use of the RMS of the signal in the CF and the mean of the absolute values for the IF. Therefore, CF and IF are defined as follows:

(5) $$\begin{array}{cc} \mathrm{CF} & =\frac{\max_{i=1,\ldots ,N}\left|x_{i}\right|}{\sqrt{\frac{1}{N}\sum_{i=1}^{N}x_{i}^{2}}}, \end{array}$$

(6) $$\begin{array}{cc} \mathrm{IF} & =\frac{\max_{i=1,\ldots ,N}\left|x_{i}\right|}{\frac{1}{N}\sum_{i=1}^{N}\left|x_{i}\right|}. \end{array}$$

It is worth highlighting the mathematical relation among SF, CF, and IF as $\mathrm{SF}=\frac{\mathrm{IF}}{\mathrm{CF}}$.

The last of the mentioned indicators, MF (also known as the clearance factor or clearance indicator), has been studied as its value is influenced when a damaged raceway is in contact with rolling elements [87,88]. It is calculated as:

(7) $$\begin{array}{c} \mathrm{MF}=\frac{\max_{i=1,\ldots ,N}\left|x_{i}\right|}{{\left(\frac{1}{N}\sum_{i=1}^{N}\sqrt{\left|x_{i}\right|}\right)}^{2}}. \end{array}$$

In addition to previous indicators, the use of statistical moments in the time domain can be found in the literature [44,89,90]. The most widely used statistic standardized moments are the variance ($\sigma^{2}$), the skewness (γ), and the kurtosis (κ). The value of $\sigma^{2}$ (or second normalized moment) can be used to measure the dispersion around the mean value of the analyzed signal, and it is obtained by:

(8) $$\begin{array}{c} \sigma^{2}=\frac{\sum_{i=1}^{N}{\left(x_{i}-\mu \right)}^{2}}{N}\;, \end{array}$$

where μ is the mean value of the analyzed signal (also known as the first normalized moment). The skewness, or the third normalized moment, quantifies the number of times that a signal value is greater or smaller than the mean of the signal. It is calculated as:

(9) $$\begin{array}{c} \gamma =\frac{\sum_{i=1}^{N}{\left(x_{i}-\mu \right)}^{3}}{N\sigma^{3}}. \end{array}$$

The fourth standardized moment, called kurtosis, is another value that is used for the analysis of a signal. The kurtosis measures the concentration of the signal around its mean value.

(10) $$\begin{array}{c} \kappa =\frac{\sum_{i=1}^{N}{\left(x_{i}-\mu \right)}^{4}}{N\sigma^{4}}. \end{array}$$

Other statistical indicators can be obtained based on the derivatives of the signal. The first derivative of the vibration signal $x^{\prime }=\left\{x_{1}^{\prime },x_{2}^{\prime },\ldots ,x_{N-1}^{\prime }\right\},\;N\in N$ is approximated as:

$$\begin{array}{c} x_{i}^{\prime }=x_{i+1}-x_{i}\;,\;i=1,\ldots ,N-1, \end{array}$$

where *N* is the length of *x*. The second derivative $x^{\prime \prime }=\left\{x_{1}^{\prime \prime },x_{2}^{\prime \prime },\ldots ,x_{N-2}^{\prime \prime }\right\},\;N\in N$ can also be approximated as:

$$\begin{array}{c} x_{i}^{\prime \prime }=x_{i+1}^{\prime }-x_{i}^{\prime }\;,\;i=1,\ldots ,N-2\;. \end{array}$$

These definitions are used by the Hjorth parameters [91], which are the activity (A), mobility (M), and complexity (C) [39,40,41]. The first activity measures the variance of the amplitude of the signal that is analyzed:

(11) $$\begin{array}{c} A_{x}=\sigma_{x}^{2}\;, \end{array}$$

where $\sigma_{x}$ is the standard deviation. The mobility is the second parameter mentioned, and measures the relative average slope of the signal:

(12) $$\begin{array}{c} M_{x}=\frac{\sqrt{A_{x^{\prime }}}}{\sqrt{A_{x}}}=\frac{\sigma_{x^{\prime }}}{\sigma_{x}}\;, \end{array}$$

where $\sigma_{x^{\prime }}$ is the standard deviation of the first derivative of the vibration signal. The complexity is the ratio of mobility and its first derivative, and it indicates how close the vibration signal to a pure sine wave resembles:

(13) $$\begin{array}{c} C_{x}=\frac{M_{x^{\prime }}}{M_{x}}=\frac{\sigma_{x^{\prime \prime }}/\sigma_{x^{\prime }}}{\sigma_{x^{\prime }}/\sigma_{x}}\;, \end{array}$$

where $\sigma_{x^{\prime \prime }}$ is the standard deviation of the second derivative of the vibration signal.

The CIs used in this study is not limited to the time domain, as indicators based on the frequency domain are also considered. Some indicators proposed in the literature, such as frequency center (FC), root mean square frequency (RMSF), and root variance frequency (RVF) are used in several works [35,92]. The first two indicators can help reveal the position change of the main frequencies of the signal, and the third indicator quantifies the convergence of the power spectrum. Their formulas are as follows:

(14) $$\begin{array}{cc} \mathrm{FC} & =\frac{\sum_{i=2}^{N-1}x_{i}^{\prime }x_{i}}{2\pi \sum_{i=1}^{N}x_{i}^{2}}, \end{array}$$

(15) $$\begin{array}{cc} \mathrm{RMSF} & =\frac{1}{2\pi }\sqrt{\frac{\sum_{i=2}^{N-1}{\left(x_{i}^{\prime }\right)}^{2}}{\sum_{i=1}^{N}x_{i}^{2}}}, \end{array}$$

(16) $$\begin{array}{cc} \mathrm{RVF} & =\sqrt{\frac{\sum_{i=2}^{N-1}{\left(x_{i}^{\prime }\right)}^{2}}{4\pi^{2}\sum_{i=1}^{N}x_{i}^{2}}-{\left(\frac{\sum_{i=2}^{N-1}x_{i}^{\prime }x_{i}}{2\pi \sum_{i=1}^{N}x_{i}^{2}}\right)}^{2}}. \end{array}$$

The 16 indicators described in Equations (1)–(16) are used in this study for the characterization of the vibration signals.

### 3.2. Entropy-Based Indicators

The indicators proposed in this work are based on the concept of entropy, which was first discussed for the second law of thermodynamics [93]. After this statement, the common understanding of entropy was associated as a measure of uncertainty, disorder, or lack of knowledge of the state of a system [94]. The idea of entropy for information measurement is conceived by Shannon [95], who calculates the entropy of messages in a communication system. Using this procedure, he explained that a regular event has less information on that system than an unlikely event. First, the set of probabilities *p* is defined as: [95]:

$$\begin{array}{c} p={\left\{p_{i}\right\}}_{i=1,\ldots ,N},p_{i}>0,\;N\in N, \end{array}$$

where $p_{i}$ is the probability that a system will be in state *i* from *N* possible states [95]. Second, the Shannon entropy *H* is defined as:

(17) $$\begin{array}{c} H=-K\sum_{i=1}^{N}p_{i}\log_{2}\left(p_{i}\right)\;, \end{array}$$

where *K* is a constant. Since Shannon based his work on a digital communication system, the base of the logarithm is 2. In general, the base of the logarithm depends on the application, with the natural and the decimal logarithm being the most commonly used bases [95].

The quantification of information related to an event from a system is the main basis for using entropy-based indicators (EIs) for the analysis of time signals in general and vibration signals, particularly for this work, as EIs attempts to measure the uncertainty or irregularity of the analyzed signal [96]. For this study, four EIs are used to extract information about the state of the bearing from the dataset described in Section 2: Approximate entropy, dispersion entropy, singular value decomposition entropy, and spectral entropy of the permutation entropy. To explain EIs, the general approach will be followed by a numerical example. For simplicity, the following sequence *x* is used in this section to provide a numerical example:

(18) $$\begin{array}{c} x={\left\{x_{i}\right\}}_{i=1,\ldots ,10}=\left\{x_{1},x_{2},\ldots ,x_{10}\right\}=\left\{6,6,9,1,9,8,7,5,2,4\right\}. \end{array}$$

#### 3.2.1. Approximate Entropy

**Figure 3:**
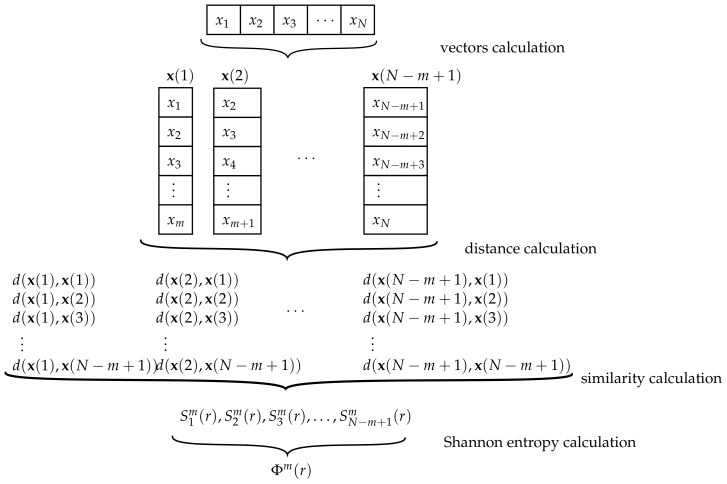
Diagram of the algorithm of approximate entropy (AppEn).

The approximate entropy (AppEn) was proposed by Pincus [97] as a family of formulas and statistics oriented to classify complex systems. After demonstrating its capabilities for time signal analysis [98], it was used for physiological signal processing [45,46,99,100]. To date, it has been used in several fields such as climate forecasts [101,102], finance [103], image encryption authentication [89,104], and fault diagnosis [105,106]. The approximate entropy (AppEn) basically quantifies the regularity and unpredictability of the analyzed signal by comparing sliding vectors from the original signal. The steps followed for the calculation of AppEn is shown in Figure 3.

The parameters involved in the AppEn calculation are *m*, which is the embedding dimension, and the tolerance value *r*. The first step is to split the analyzed signal of length *N* into N−m+1 vectors x(i) having m∈N consecutive elements:

(19) $$x(i)\;=\left(x_{i},x_{i+1},\ldots ,x_{i+m-1}\right),\;i=1,\ldots ,N-m+1.$$

For the proposed sequence *x*, using m=3, the first vector sequence is x(1)=(6,6,9). Subsequently, the second vector sequence is x(2)=(6,9,1) and the third x(3)=(9,1,9). In total, eight x(i) trios can be built. These are:

$$\begin{array}{c} \begin{array}{cc} X & =\{x(1),x(2),\ldots ,x(8)\}\\ \; & =\left\{(6,6,9),(6,9,1),(9,1,9),(1,9,8),(9,8,7),(8,7,5),(7,5,2),(5,2,4)\right\}. \end{array} \end{array}$$

Secondly, a metric distance *d* is defined to compare two vectors x(i) and x(j) as the maximum absolute difference between two respective scalar values after comparing the values from all elements of the vectors [97]:

(20) $$\begin{array}{c} d\left(x\left(i\right),x\left(j\right)\right)=\max_{k=0,\ldots ,m-1}\left|x_{i+k}-x_{j+k}\right|,\;i,j=1,\ldots ,N-m+1. \end{array}$$

The proposed distance is calculated for all possible pair vectors x(i) and x(j). A clear consequence of this definition is that the distance from one vector to itself is zero. For the proposed example, the distance between x(1) and x(2) is calculated as follows:

$$\begin{array}{c} \begin{array}{cc} d(x(1),x(2)) & =\max\{|x_{1}-x_{2}|,|x_{2}-x_{3}|,|x_{3}-x_{4}|\}\\ \; & =\max\{|6-6|,|6-9|,|9-1|\}\\ \; & =8. \end{array} \end{array}$$

The same process is performed to compare x(1) with all other vectors (itself included). The calculated values are:

$$\begin{array}{cccccccc} d(x(1),x(1)) & =0, & d(x(1),x(2)) & =8, & d(x(1),x(3)) & =5, & d(x(1),x(4)) & =5,\\ d(x(1),x(5)) & =3, & d(x(1),x(6)) & =4, & d(x(1),x(7)) & =7, & d(x(1),x(8)) & =5. \end{array}$$

As the calculation process is performed for all pair vectors, the proposed example ends with the calculation of the distances between x(8) and x(j),j=1,…,8.

After the distances among vectors are met, the next step is the calculation of the similarity, which is unique for each vector. The correlation dimension of a vector, or similarity, is calculated as the division of the number of times that the distance between the vectors x(i) and x(j),j=1,…,N−m+1 is less than or equal to the tolerance parameter *r*, and N−m+1:

(21) $$\begin{array}{cc} S_{i}^{m}\left(r\right) & =\frac{1}{N-m+1}\sum_{j=1}^{N-m+1}s_{ij}\;, \end{array}$$

where

$$\begin{array}{cc} 1-1s_{ij} & =\left\{\begin{array}{cc} 1, & \;\mathrm{if}\;\;d(x(i),x(j))\leq r\\ 0, & \;\mathrm{otherwise}\; \end{array}\right.. \end{array}$$

From the proposed example, with the use of r=5, the similarity values of all vectors are:

$$\begin{array}{cccccccc} S_{1}^{3}\left(5\right) & =\frac{5}{8}, & \;S_{2}^{3}\left(5\right) & =\frac{1}{4}, & \;S_{3}^{3}\left(5\right) & =\frac{1}{4}, & \;S_{4}^{3}\left(5\right) & =\frac{1}{8},\\ S_{5}^{3}\left(5\right) & =\frac{3}{8}, & S_{6}^{3}\left(5\right) & =\frac{5}{8}, & S_{7}^{3}\left(5\right) & =\frac{1}{2}, & S_{8}^{3}\left(5\right) & =\frac{1}{2}. \end{array}$$

The penultimate step is the Shannon entropy calculation for all $S_{i}^{m}\left(r\right)$, which is defined as the averaged sum of the natural logarithm of the similarities:

(22) $$\begin{array}{c} \Phi^{m}\left(r\right)=\frac{1}{N-m+1}\sum_{\begin{array}{c} i=1\\ S_{i}^{m}\left(r\right)>0 \end{array}}^{N-m+1}\ln\left(S_{i}^{m}\left(r\right)\right). \end{array}$$

The AppEn is the limit of the difference between $\Phi^{m}\left(r\right)$ and $\Phi^{m+1}\left(r\right)$, where $\Phi^{m+1}\left(r\right)$ is the Shannon entropy from all $S_{i}^{m+1}\left(r\right)$ values [97]:

$$\begin{array}{c} \mathrm{AppEn}\left(m,r\right)=\lim_{N\to +\infty }\left[\Phi^{m}\left(r\right)-\Phi^{m+1}\left(r\right)\right]. \end{array}$$

Since the analysis is performed on a time signal of finite length *N*, AppEn is estimated by the definition of the statistic indicator [107]:

(23) $$\begin{array}{c} \mathrm{AppEn}\left(m,r,N\right)=\Phi^{m}\left(r\right)-\Phi^{m+1}\left(r\right). \end{array}$$

As m=3 and r=5 for the proposed example, Equation (23) is calculated by the use of $\Phi^{3}\left(5\right)$ and $\Phi^{4}\left(5\right)$:

$$\begin{array}{cc} \mathrm{AppEn}(3,5,10) & =\Phi^{3}\left(5\right)-\Phi^{4}\left(5\right)\approx -1.019895-\left(-1.079734\right)\approx 0.059839. \end{array}$$

The AppEn evaluates the regularity of the signal, with the regularity defined as the tendency for the signal to have recurrent patterns throughout the signal [108]. Using this idea, the values of AppEn would be lower for regular signals and higher for complex signals. For example, a periodic vibration signal would have a low AppEn value (close to zero) because of the regularity (or periodicity) of the signal. In contrast, owing to its lower regulariy, a complex vibration signal with multiple frequency components will have a higher AppEn value compared to a periodic vibration signal.

#### 3.2.2. Dispersion Entropy

**Figure 4:**
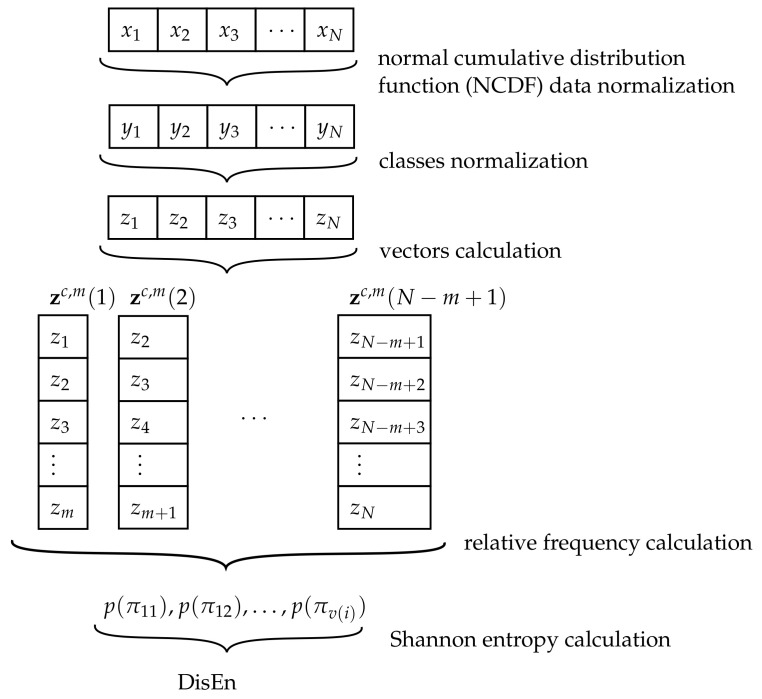
Diagram of the algorithm of dispersion entropy (DisEn).

The next EI to be described is the dispersion entropy (DisEn), which was first proposed by Rostaghi et al. [96] as an alternative to the sample and permutation entropy [46,49,100,109]. Although this indicator is relatively new in comparison to AppEn, DisEn has gained popularity in the analysis of biomedical signals [48,110,111,112], economics [50], authentication processes [52], and rotary machine fault detection [113,114,115]. The steps followed for the calculation of DisEn is shown in Figure 4.

The calculation of DisEn starts with the parameters *c* and *m*, where c∈N is the number of classes and m∈N is the embedding dimension. First, the values of the analyzed signal are normalized using a normal cumulative distribution function (NCDF) to later create several embedding vectors and obtain dispersion pattern indicators. Finally, based on the Shannon entropy definition (Equation (34)), the DisEn value is obtained.

As previously mentioned, the first step consists of the normalization of the values *x* into $y=\{y_{1},y_{2},\ldots ,y_{N}\}$ using an NCDF to set the values between 0 and 1 [116]:

(24) $$\begin{array}{c} y_{i}=\frac{1}{\sigma \sqrt{2\pi }}\int_{-\infty }^{x_{i}}e^{\frac{-{\left(t-\mu \right)}^{2}}{2\sigma^{2}}}dt \end{array}$$

where μ and σ are the mean and standard deviation of the signal, respectively.

For the proposed example, this calculation gives the sequence *y* as follows:

$$\begin{array}{c} y={\left\{y_{i}\right\}}_{i=1,\ldots ,10}=\left\{y_{1},y_{2},\ldots ,y_{10}\right\}=\left\{0.54,0.54,0.89,0.03,0.89,0.81,0.69,0.39,0.07,0.25\right\}. \end{array}$$

Thereafter, the values of *y* are classified into c≥2 classes using the formula:

(25) $$\begin{array}{c} z_{i}^{c}=\mathrm{round}\left(c\;\cdot \;y_{i}+0.5\right). \end{array}$$

For the numerical example proposed using the sequence *x*, the values of $z_{i}^{c}$ are:

$$\begin{array}{c} z^{3}=\left\{z_{i}^{3}\right\}=\left\{2,2,3,1,3,3,3,2,1,1\right\}. \end{array}$$

The following step is to create the embedding vectors $z^{c,m}\left(i\right)$ with embedding dimension *m*, where a total of N−m+1 vectors can be calculated:

(26) $$\begin{array}{cc} Z^{c,m} & =\{z^{c,m}\left(1\right),z^{c,m}\left(2\right),\ldots ,z^{c,m}\left(N-m+1\right)\}, \end{array}$$

where

(27) $$\begin{array}{cc} z^{c,m}\left(i\right) & =\left(z_{i}^{c},z_{i+1}^{c},\ldots ,z_{i+m-1}^{c}\right),\;i=1,\ldots ,N-m+1. \end{array}$$

Inside $z_{m}^{c}\left(i\right)$, the order of values can follow certain patterns. These patterns are denoted as $\pi_{v_{1}v_{2}\cdots v_{m}}$, where $1\leq v_{i}\leq c,\;i=1,\ldots ,m$. The maximum number of patterns is $c^{m}$, which is the number of variations with repetition $VR^{c,m}$.

For the example in this work, the value of the embedding dimensions is m=2, and the length of the sequence *x* is N=10. Therefore, N−m+1=9 vectors can be created, which are:

$$\begin{array}{cc} Z^{3,2} & =\{z^{3,2}\left(1\right),z^{3,2}\left(2\right),\ldots ,z^{3,2}\left(9\right)\}\\ \; & =\{(2,2),(2,3),(3,1),(1,3),(3,3),(3,3),(3,2),(2,1),(1,1)\}. \end{array}$$

In the numerical example, the maximum number of patterns is $3^{2}=9$.

For the calculation of DisEn, the relative frequency of each pattern is needed. This frequency is calculated as the number of times that a pattern is present on $Z^{c,m}$, divided by the total number of possible dispersion patterns $c^{m}$:

(28) $$\begin{array}{c} p\left(\pi_{v_{1}v_{2}\ldots v_{m}}\right)=\frac{\#\{i\in \left\{1,\ldots ,N-m+1\right\}\;|\;z^{c,m}\left(i\right)\;\mathrm{has}\;\mathrm{type}\;\pi_{v_{1}v_{2}\ldots v_{m}}\}}{N-m+1}. \end{array}$$

In the proposed example because one pattern is not present, eight pattern values are obtained:

$$\begin{array}{cccccccc} p(\pi_{11}) & =\frac{1}{9}, & \;p(\pi_{13}) & =\frac{1}{9}, & \;p(\pi_{21}) & =\frac{1}{9}, & \;p(\pi_{22}) & =\frac{1}{9},\\ \;p(\pi_{23}) & =\frac{1}{9}, & \;p(\pi_{31}) & =\frac{1}{9}, & \;p(\pi_{32}) & =\frac{1}{9}, & \;p(\pi_{33}) & =\frac{2}{9}\;. \end{array}$$

The last step is the use of the definition of the Shannon entropy with the frequencies calculated to obtain DisEn, where only the patterns $p(\pi_{v_{1}v_{2}\ldots v_{m}})>0$ have been considered [95]:

(29) $$\begin{array}{c} \mathrm{DisEn}\left(c,m\right)=-\sum_{\begin{array}{c} i=1\\ p(\pi_{v(i)})>0 \end{array}}^{c^{m}}p\left(\pi_{v(i)}\right)\ln\left(p\left(\pi_{v(i)}\right)\right), \end{array}$$

where v(i) is the *i*-th variation with the repetition of *m* elements from the set {1,2,…,c}.

For the case of the numerical example, the calculation of DisEn is:

$$\begin{array}{c} \mathrm{DisEn}\left(3,2\right)=-7\frac{1}{9}\ln\left(\frac{1}{9}\right)+\frac{2}{9}\ln\left(\frac{2}{9}\right)\approx 2.043191. \end{array}$$

This value can be normalized by dividing DisEn by $\ln(c^{m})$. Hence, the normalized DisEn (NDisEn) is defined as:

(30) $$\begin{array}{c} \mathrm{NDisEn}=\frac{\mathrm{DisEn}}{\ln(c^{m})}. \end{array}$$

For the proposed example, the value of NDisEn is:

$$\begin{array}{c} \mathrm{NDisEn}=\frac{-7\frac{1}{9}\ln\left(\frac{1}{9}\right)+\frac{2}{9}\ln\left(\frac{2}{9}\right)}{\ln(9)}\approx 0.929896. \end{array}$$

The value of DisEn is higher when all the possible dispersion patterns have equal frequency values on a signal thus, an irregular signal is present. However, the presence of fewer patterns will result from lower values of DisEn thus, a completely regular signal is obtained [96]. DisEn is relatively insensitive to noise because larger or smaller changes in the amplitude of the signal do not vary significantly the class label of the values [117].

#### 3.2.3. Single Value Decomposition Entropy

**Figure 5:**
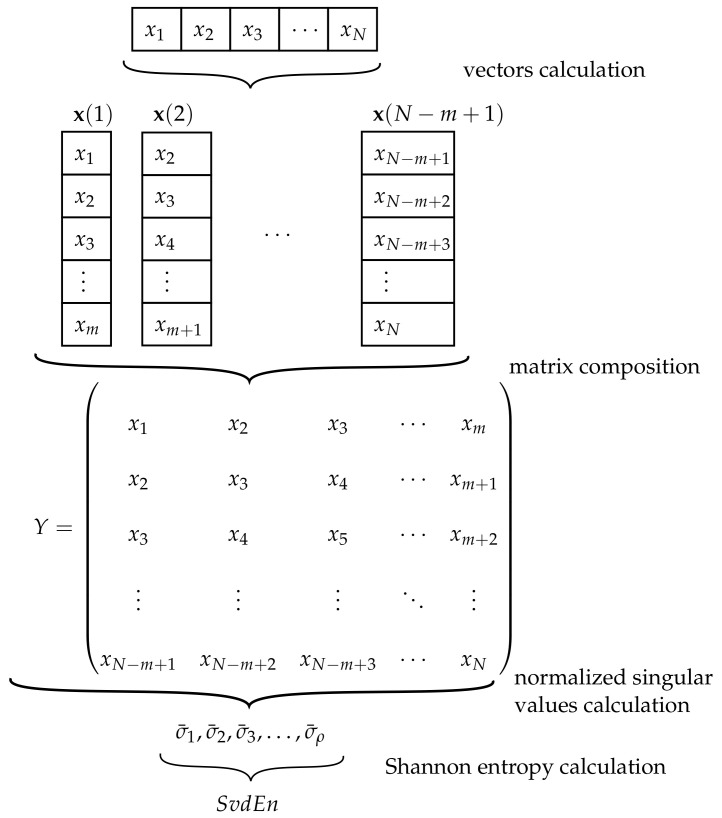
Diagram of the singular value decomposition entropy (SvdEn) computation.

The third EI used in this study is the singular value decomposition entropy (SvdEn). This entropy calculation, which was proposed by Roberts et al. [118], was an attempt to improve the data analysis from an electroencephalogram (EEG), especially for cases where movements are thought by patients but are not performed in reality. Compared with AppEn and DisEn, SvdEn are used mostly in EEG analysis [119,120], and medical imaging [121,122]. The steps followed for the computation of SvdEn is shown in Figure 5.

The idea behind SvdEn is to indicate the number of eigenvectors needed for an adequate explanation of the analyzed signal, giving an idea of its dimensionality and complexity. Therefore, the only parameter involved in the SvdEn calculation is the embedding dimension *m*. To understand the procedure, the numerical calculation of SvdEn is presented with the sequence *x* defined in Equation (18).

First, the creation of embedding vectors x(i) of consecutive m∈N values is needed. The procedure is similar to the vectors needed in AppEn and DisEn:

(31) $$\begin{array}{c} X=\{x(1),x(2),\ldots ,x(N-m+1)\}. \end{array}$$

For the case of the numerical example of the sequence *x* and considering the parameter value m=5, the values of x(i) are as follows:

$$\begin{array}{c} X=\left\{(6,6,9,1,9),(6,9,1,9,8),(9,1,9,8,7),(1,9,8,7,5),(9,8,7,5,2),(8,7,5,2,4)\right\}. \end{array}$$

The subsequent action is the composition of the matrix *Y*, which is built based on the vectors obtained from x(i) in Equation (31):

(32) $$\begin{array}{c} Y=\left(\begin{array}{c} x(1)\\ x(2)\\ \vdots \\ x(N-m+1) \end{array}\right)\in M_{(N-m+1)\times m}\left(R\right). \end{array}$$

For the example considered in this paper, the matrix *Y* is stated as:

$$\begin{array}{c} Y=\left(\begin{array}{ccccc} 6 & 6 & 9 & 1 & 9\\ 6 & 9 & 1 & 9 & 8\\ 9 & 1 & 9 & 8 & 7\\ 1 & 9 & 8 & 7 & 5\\ 9 & 8 & 7 & 5 & 2\\ 8 & 7 & 5 & 2 & 4 \end{array}\right). \end{array}$$

Once the matrix *Y* is built, the next step is the calculation of the ρ singular values $\sigma =\{\sigma_{1},\ldots ,\sigma_{\rho }\}$ of matrix *Y*, assuming that ρ<N−m+1 and rank(Y)=ρ. Once the values are obtained, the normalized singular values $\bar{\sigma }=\left\{{\bar{\sigma }}_{1},\ldots ,{\bar{\sigma }}_{\rho }\right\}$ are:

(33) $$\begin{array}{c} {\bar{\sigma }}_{i}=\frac{\sigma_{i}}{\sum_{j=1}^{\rho }\sigma_{j}},\;i=1,\ldots ,\rho . \end{array}$$

In the numerical example, the ${\bar{\sigma }}_{i}$ values from *Y* are as follows:

$$\begin{array}{c} \bar{\sigma }=\left\{0.537047,0.145508,0.116931,0.109984,0.0905299\right\}. \end{array}$$

The final step, as in the rest of the previous EIs, is the use of the Shannon entropy definition on the normalized $\bar{\sigma }$ values, and with the use of base 2 for the logarithm [118]:

(34) $$\begin{array}{c} \mathrm{SvdEn}\left(\rho \right)=-\sum_{\begin{array}{c} i=1\\ {\bar{\sigma }}_{i}>0 \end{array}}^{\rho }{\bar{\sigma }}_{i}\;\cdot \;\log_{2}\left({\bar{\sigma }}_{i}\right). \end{array}$$

This final step can be translated to the numerical example for the proposed sequence *x*, with the following result:

$$\begin{array}{c} \mathrm{SvdEn}\left(5\right)=-\sum_{i=1}^{5}{\bar{\sigma }}_{i}\;\cdot \;\log_{2}\left({\bar{\sigma }}_{i}\right)\approx 1.912336. \end{array}$$

In addition, as in the case of DisEn, the SvdEn values can be normalized, and the normalized SvdEn (NSvdEn) is defined. The step is similar to the mentioned EI, but the base of the logarithm *e* is changed to 2, and $c^{\rho }$ is replaced by ρ, leading to:

(35) $$\begin{array}{c} \mathrm{NSvdEn}=\frac{\mathrm{SvdEn}(\rho )}{\log_{2}\left(\rho \right)}. \end{array}$$

For the proposed numerical example, the value of NSvdEn is:

$$\begin{array}{c} \mathrm{NSvdEn}=\frac{\mathrm{SvdEn}(5)}{\log_{2}\left(5\right)}\approx 0.823598. \end{array}$$

#### 3.2.4. Spectral Entropy of the Permutation Entropy Signal

**Figure 6:**
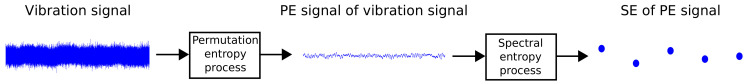
Diagram of the signal analysis procedure for spectral entropy of the permutation entropy signal SepEn [74].

The last EI used in this study is spectral entropy of the permutation entropy signal (SepEn). This indicator is defined by Sandoval et al. [74], and it is specifically designed (but not limited to) for low-speed bearings. TheSepEn uses EIs for its process: permutation entropy (PerEn) and pectral 215 entropy (SpeEn). The steps followed for the computation of SepEn is shown in Figure 6 [74].

The first step in calculating SepEn involves estimating PerEn, which starts grouping *m* consecutive values of the analyzed signal into N−m+1 groups [109], where *N* is the length of *x*:

(36) $$\begin{array}{cc} x & =\{x_{i},x_{i+1},\ldots ,x_{i+m-1}\},\;i=1,\ldots ,N-m+1. \end{array}$$

For the case of the defined sequence *x* and considering m=2, the groups are as follows:

$$\begin{array}{cc} X & =\{x(1),x(2),\ldots ,x(8)\}\\ \; & =\left\{(6,6),(6,9),(9,1),(1,9),(9,8),(8,7),(7,5),(5,2),(2,4)\right\}. \end{array}$$

Then, the permutation patterns present on the grouped values are counted. As equal consecutive values are not considered in the calculation [109], in the proposed example, only two potential permutation patterns $\pi_{j}$ have been considered: $x_{i}<x_{i+1}$ and $x_{i}>x_{i+1}$, which correspond to $\pi_{1}$ and $\pi_{2}$, respectively. Therefore, the relative frequency for each permutation pattern was calculated as:

(37) $$\begin{array}{cc} p(\pi_{j}) & =\frac{\#\left\{i\in \left\{1,\ldots ,N-m+1\right\}\;|\;x\left(i\right)\mathrm{has}\mathrm{type}\pi_{j}\right\}}{N-m+1},\;j=1,\ldots ,m!\;, \end{array}$$

obtaining $p(\pi_{1})=3/9$ and $p(\pi_{2})=5/9$ for the numerical example with *x*. Finally, the Shannon entropy is applied to the relative frequencies $p(\pi_{j}),\;j=1,\ldots ,m!$ to calculate the PerEn value:

(38) $$\begin{array}{cc} \mathrm{PerEn}(m) & =-\sum_{\begin{array}{c} j=1\\ p(\pi_{j})>0 \end{array}}^{m!}p\left(\pi_{j}\right)\log_{2}p\left(\pi_{j}\right). \end{array}$$

The value for the numerical example is:

$$\begin{array}{cc} \mathrm{PerEn}(3) & =-\frac{3}{9}\log_{2}\left(\frac{3}{9}\right)+\frac{5}{9}\log_{2}\left(\frac{5}{9}\right)\approx 0.99943. \end{array}$$

As shown in Equation (38), PerEn provides a scalar value. SepEn requires the generation of a signal based on PerEn, which is calculated using a certain data window. If the data window moves forward one data set at a time on the analyzed signal, a set of values from the window slices on the entire signal can be stated, which is called the permutation signal.

The next step in the calculation of spectral entropy of the SepEn involves SpeEn, which uses the Shannon entropy of the normalized power spectral density (PSD) of a signal. The normalized PSD of a signal is defined as follows [123]:

(39) $$\begin{array}{c} \int_{f=0}^{f_{s}/2}P\left(f\right)df=1\;, \end{array}$$

where $f_{s}$ is the sampling frequency of the analyzed signal, and P(f) is the power value of the harmonic *f*. The power of the analyzed signal is normalized to obtain the sum of the power of each harmonic as 1. Then, the SpeEn for an analyzed signal is:

(40) $$\begin{array}{cc} \mathrm{SpeEn} & =-\sum_{f=0}^{f_{s}/2}P\left(f\right)\log_{2}P\left(f\right), \end{array}$$

where the base of the logarithm is 2. As the sequence *x* is not suitable for explaining the SpeEn, the case of two signals g(t) and h(t) is proposed. The first signal g(t) is a pure sine wave with frequency $f_{a}$, and h(t) is the sum of two sine waves with frequency $f_{b}$ and $f_{c}$. While each sine wave from h(t) has a unique frequency, both the power contributions to h(t) are the same. Hence, the signals are given as follows:

$$\begin{array}{c} \begin{array}{cc} g(t) & =\sin(f_{a}t),\\ h(t) & =\sin\left(f_{b}t\right)+\sin\left(f_{c}t\right). \end{array} \end{array}$$

In the case of the signal g(t), the power of the signal is concentrated only in one frequency $f_{a}$ hence, $P(f_{a})=1$. Therefore, SpeEn is given as:

$$\begin{array}{c} \mathrm{SpeEn}=-1\;\cdot \;\log_{2}\left(1\right)=0. \end{array}$$

The case of h(t) differs from g(t) because the power of h(t) is split between the frequencies $f_{b}$ and $f_{c}$. For this reason, $P\left(f_{b}\right)=P\left(f_{c}\right)=1/2$. Consequently, the SpeEn value is:

$$\mathrm{SpeEn}=-2\;\cdot \;\left(\frac{1}{2}\right)\log_{2}\left(\frac{1}{2}\right)=1.$$

As the PSD of the signal g(t) is concentrated only in one frequency, g(t) is predictable. A different situation is stated with signal h(t), where a greater irregularity in time is present compared with g(t). Therefore, the SpeEn of h(t) is higher than g(t).

The calculation process of SepEn is summarized in the following steps:

1. The permutation entropy (PerEn) is calculated for the analyzed signal [109]. Each value is obtained for a specific time window thus, a PerEn signal is achieved by sliding the time window along the analyzed signal;
2. Using the signal calculated in the previous step, for a time window equivalent to one rotation of the bearing, the SpeEn value is acquired [124].

The validity of this EI is proven by Sandoval et al. [74], who showed that the values of spectral entropy of the SepEn for healthy bearings are higher than the values obtained from vibration signals of bearings with damage.

## 4. Methodology

In this section, the proposed diagnosis strategy is first discussed. Secondly, the parameter values used to calculate the different indicators are studied. Since CIs do not depend on parameters, entropy-based indicators (EIs) is the first to be described. Finally, Section 4.3 provides the classification algorithm used in this work, which is briefly reviewed for manuscript completeness.

### 4.1. Proposed Diagnosis Method

**Figure 7:**
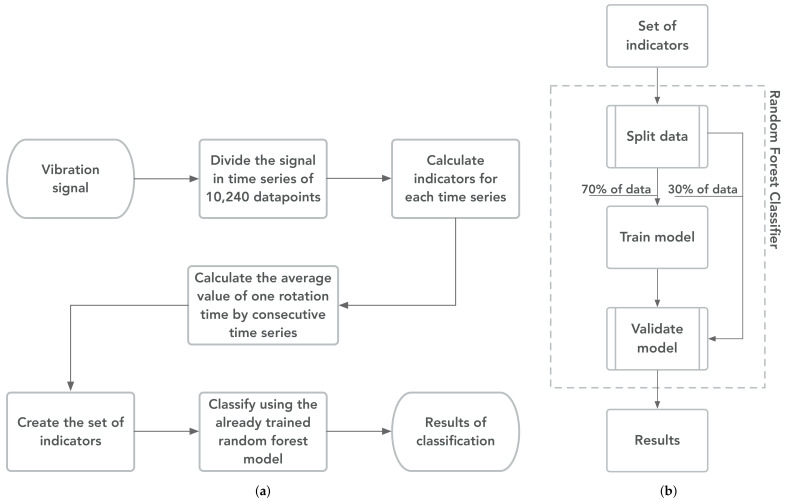
Diagram of proposed fault diagnosis method for vibration signals of low-speed bearings. (**a**) Values for general method. (**b**) Diagram to detail the training and validation of the random forest model.

This paper presents a scheme in which several low-speed bearings with induced damage are classified as a feasibility study for the classification and diagnosis of low-speed bearings based on entropy-based indicators. The classification is based on the dataset presented in Section 2. First, information is extracted from the vibration signals using four EIs, which are AppEn, DisEn, SvdEn, and SpeEn. Afterward, the extracted information is used by RF the models. The steps of the proposed classification method are summarized in Figure 7.

First, the vibration signals are split into a time series of 10,240 data points (one second). For each time series, the indicators were calculated to extract information from the data. Then, the analysis is performed for each entire rotation, where each rotation has a set of indicators. The value per indicator is calculated as the average of the indicators from the number of consecutive time series from the vibration signals required to represent the time of one rotation of the shaft washer, which is dependent on the rotational speed. For example, the AppEn of one rotation from a signal at 10 rpm is the average of 6 AppEn values. Finally, the indicators from each rotation are used as features to train and validate the RF model.

The presented scheme allows the possibility of switching the indicator group, hence performing a comparative study between EIs and CIs. A third group, called classic and entropy indicatorss (CEIs), is included to analyze the improvement of the classification by joining EIs and CIs. As the data set includes three speeds, and three indicators groups are used, nine RF models are trained.

### 4.2. Entropy-Based Indicators

The EIs is defined using multiple parameters that are selected according to the intended purpose and results. In this work, the parameter values were selected after an exhaustive study. For explanation purposes, this section uses five signals of one minute long at the same rotational speed. Each signal represents one scenario, as described in Section 2. Although the numerical results for each EI are calculated with different signal groups, the chosen group captures the general results of the explained EI.

#### 4.2.1. Approximate Entropy

**Figure 8:**
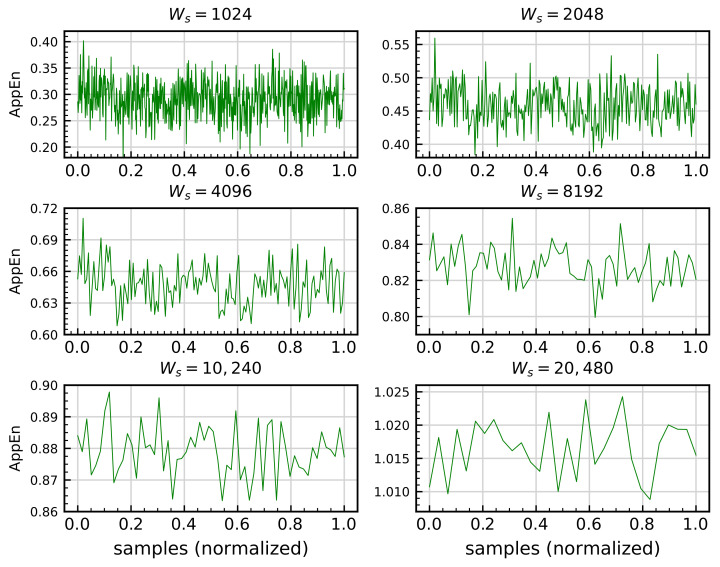
Approximate entropy (AppEn) calculation for bearing signal corresponding to healthy scenario HS with m=5, r=0.2×σ, and varying $W_{s}$.

As explained in Section 3.2.1, the calculation of AppEn involves two parameters: *m* and *r*, as shown in Equation (23). After consulting the literature, *m* values of between 3 and 10 are regularly used [42,125] thus, this range is used in this work as the initial range where the *m* value is sought. To analyze this parameter and its influence on the results, five signals per minute are used to compare the behavior of AppEn in different scenarios. The analysis starts by dividing each signal into a time series of $W_{s}=$10,240 samples, which corresponds to one second. This value is selected after comparing several values of $W_{s}$. For example, Figure 8 shows a comparison of the AppEn calculation for one HS signal using different $W_{s}$.

The results reveal that for values lower than $W_{s}=$10,240, a larger amount of noise and the loss of any representativeness of the original signal can be observed in the calculated signal. The same procedure is accomplished for all EIs with the same results. Therefore, $W_{s}=$10,240, is used in all EIs calculations.

**Figure 9:**
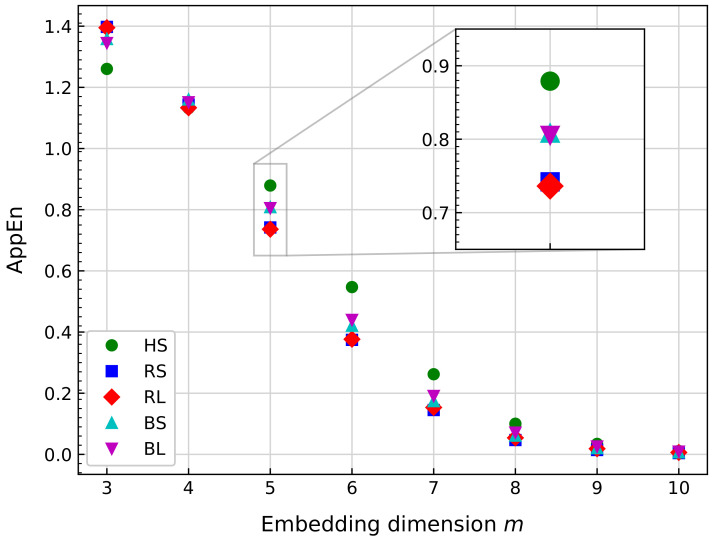
Comparison of the approximate entropy (AppEn) mean value for several bearing signals at the same rotational speed. The calculation uses r=0.2×σ, $W_{s}=$ 10,240, and varying *m*.

For each time series, AppEn is calculated. Finally, the average of the AppEn values for the entire signal is obtained and compared with the values from other signals. Figure 9 shows several AppEn values obtained by varying *m* from 3 to 10 with a fixed r=0.2×σ (where σ is the standard deviation of the signal).

From Figure 9, there is no clear difference among scenarios for m=4, 8, 9, and 10. For m=3,5,6, and 7, HS can be easily discriminated from other scenarios. In addition, approximate entropy (AppEn) can differentiate scenarios by damage location for m=3,5,6. In the case of the damage size, Figure 9 shows that AppEn is unable to make a difference in the results for RS and RL, as well as for BS and BL. As the damage location is the only attribute where AppEn can draw a distinction, and to maximize this difference, the value of m=5 is selected.

With respect to the *r* parameter, this value helps to filter the information obtained from the signal [108]. The problem with this parameter from a mathematical point of view has been detailed previously [98], and it is later calculated as a dependent value of σ from the analyzed signal [42,106,125]. Empirically, the nature of the vibration signals of the dataset means that no value of r<0.5×σ affects the calculation, and r=0.2×σ is one of the values selected normally in the literature. Therefore, in this work, r=0.2×σ was used to obtain the results.

#### 4.2.2. Dispersion Entropy

**Figure 10:**
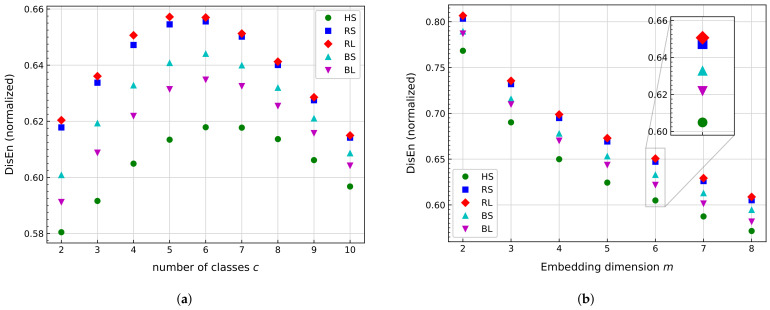
Comparison of dispersion entropy (DisEn) mean value for several bearing signals at same rotational speed, with fixed $W_{s}=$ 10,240. (**a**) Values for fixed *m* = 6, varying *c*. (**b**) Values for fixed c = 4, varying *m*.

As stated in Section 3.2.2, the DisEn indicator depends on the parameters *c* and *m*; see Equation (29). From the literature [126,127], the value of *c* is usually between 2 and 10, and *m* is no further than 5. Therefore, these ranges are studied for the specific application of low-speed bearing vibration signals. Consequently, the parameter values are calculated using the same methodology that was employed for the approximate entropy (AppEn): Five signals per minute are split on a time series of $W_{s}=10240$ data points. The indicator is calculated for each time series, and later, the average is obtained to contrast all signals. Figure 10 shows the results obtained with a fixed m=6 varying *c* and fixed c=4 varying *m*.

The results show that for both parameters, HS can be distinguished from other scenarios. Figure 10a illustrates that for all *c* values, HS has the smallest value from all signals. Based on a damage localization perspective, DisEn can be used to differentiate the cases from the ball or raceway damage scenario, but this difference decreases for c>5. In addition, from Figure 10a, the damage size can be clearly distinguished for the ball damage scenario, yet it is somewhat less for the raceway damage scenario. To maximize the difference among scenarios, c=2,3,4, and 5 are good candidates.

As DisEn cannot discriminate RS and RL as well as BS and BL, the major difference in value between BS and BL is noted for c=4. In addition, the difference in value between HS and all other scenarios is higher for c=4 compared with c=2,3, and 5. Therefore, the value selected for *c* was 4.

Subsequently, Figure 10b shows the results of DisEn for variable *m*, with fixed c=4. Similar to Figure 10a, HS has the lowest values for all cases of *m*. As damage scenarios can be distinguished for all values of DisEn, RS and RL are not easily distinguished. A progressive difference between BS and BL is stated as *m* increases. Since $c^{m}$ should be less than $W_{s}$, [126], the selected value is m=6.

#### 4.2.3. Single Value Decomposition Entropy

**Figure 11:**
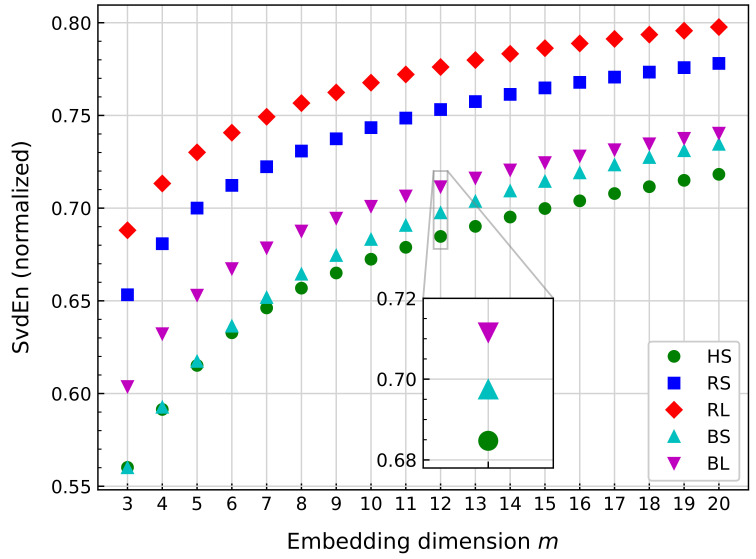
Comparison of the single value decomposition entropy (SvdEn) mean value for several bearing signals at the same rotational speed, with fixed $W_{s}=$10,240.

As stated in Section 3.2.3, SvdEn depends on one parameter, *m*, as shown in Equation (34). In the case of medical imaging and EEG analysis, *m* is usually selected between the range 5–10 [47,128]. As vibration signals are quite different from EEG signals, further analysis is performed. Figure 11 summarizes the influence of the parameter on the obtained SvdEn for all the rotational speeds, where five signals of a one minute length are analyzed, with $W_{s}=$10,240.

From Figure 11, the data show how SvdEn cannot establish a distinction among scenarios for values m<7 because the HS and BS values overlap. For m≥7, the lower values for each embedding dimension *m* correspond to HS. From the figure, SvdEn can supply a difference between HS and scenarios RS and RL for all values of *m*. In the case of BS and BL, a visible separation from HS is observed for m>7. Another situation is observed for BS values, which are closer to HS for lower values of *m*. As *m* increases, the BS values are closer to BL than BS. For m=12, the value difference of BS to HS and BL is a maximum compared to all other values of *m*. Therefore, this value is selected.

#### 4.2.4. Spectral Entropy of Permutation Entropy

**Figure 12:**
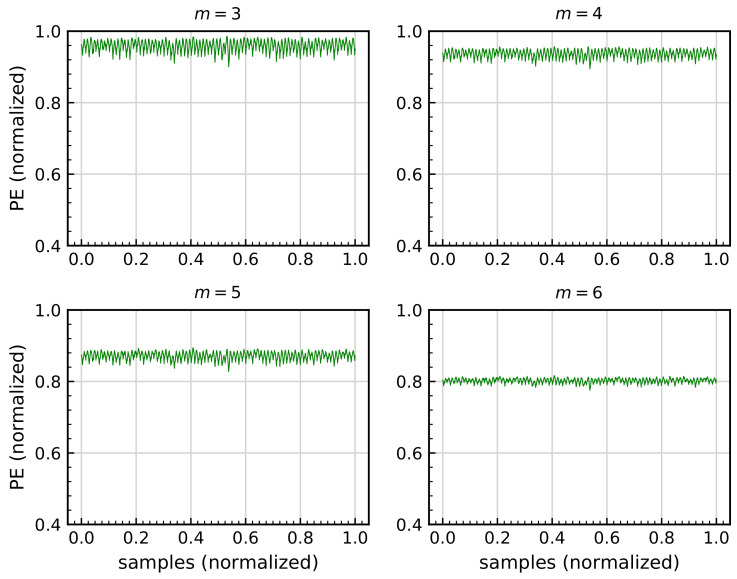
Comparison of the permutation entropy PerEn signal calculated for 30 s of a large raceway shaft washer damage scenario RL signal, with fixed $W_{s}=$10,240.

As stated in Section 3.2.4, spectral entropy of the permutation entropy signal (SepEn) depend on one parameter, *m*, as shown in Equation (36). According to previous work [74], suitable values for *m* can range from 3 to 6, with similar results. Since the use of a higher value only implies more computational effort, the value m=3 is used. This can be viewed in Figure 12, where 30 s of the same signal RL is used for comparison purposes.

In contrast with the previous EIs, the values from SpeEn correspond to each rotation made by the shaft washer. To obtain the value of each rotation, the average of each $W_{s}=$10,240 is calculated [74]. This limits the use of all previous indicators to have only one value per rotation, as SpeEn does.

### 4.3. Random Forest

The RF algorithm is used for damage classification. The first concept of RF was introduced by Ho [129] and later by Breiman [130], who gave controlled variance to the algorithm proposed by Ho. RF is an ensemble learning model that improves the capabilities of the decision tree (DT) classifier [131] by bagging ensemble learning [132]. In this respect, RF uses several DTs to decide the classification by means of decisions according to the majority of DTs. This explains the number of parameters to be determined by the RF algorithm, which starts with the parameters for the DT algorithm [131], in addition to the number of trees.

In summary, the following steps describe the operation of the RF classifier:

1. Set the parameters for RF, which include the intrinsic parameters for DT, and the number of trees to employ;
2. After the number of DT is established, a stratified sampling of the data set is set as train and test data. The proportion between both groups of data is fixed for all DT, but the elements of each data group are selected randomly [129];
3. Each DT classifies the data using a random feature from the train data, to arbitrarily classify and increase the difference between DTs. This step is fundamental because it improves the generalization error;
4. The results of all DT enable the classification of the test samples to be determined using the vote of each DT. The decision is done by majority voting.

As mentioned, the RF algorithm is an ensemble machine learning technique that has various hyper-parameters inherited from the decision tree (DT) algorithm. The hyper-parameters are those parameters that are intrinsic to the technique, whose values cannot be estimated from the data [133]. To select the value of a hyper-parameter, one recurrent process is to determine the value using experimental results [134].

For RF, the hyper-parameters inherited from DT are:

- The function criteria to measure the quality of a split, which is set as the Gini impurity;
- The maximum depth of the tree, which is set to three for this work;
- The maximum number of leafs, which is unlimited;
- The minimum number of samples required to split an internal node, which is two;
- The minimum number of samples required to be at a leaf node, which is one.

Another hyper-parameter from RF is the number of trees used for the model. Since no further changes in the results above 100 trees were observed, this value was used. Once the hyper-parameters of the RF algorithm are set, the data are split by the hold-out method for validation. A RF model is trained on 70% of the total data, and the rest of the data are used to calculate the classification accuracy score [135]. This process is repeated 1000 times for the validation of the trained models based on the indicator groups, where train and test groups are created with randomly selected data. Finally, the classification accuracy scores are averaged as representative results.

## 5. Results and Discussion

This section shows the accuracy scores obtained from the RF classifier trained models based on the dataset presented in Section 2 by using the indicators mentioned in Section 3. As the calculation is based on the number of rotations, Table 1 shows the number of rotations available for each scenario and rotational speed.

As previously mentioned, three indicator groups are used in this study: CIs, entropy-based indicators (EIs), and CEIs. As the RF classifier chooses the training and testing data randomly, this process is repeated 1000 times to obtain representative results. The classification accuracy scores for the trained models are summarized in Table 2, where two columns can be found for each indicator group: The first represents the averaged accuracy scores (${\bar{x}}_{\mathrm{as}}$), and the second is the standard deviation of the accuracy scores ($\sigma_{\mathrm{as}}$) of the 1000 combinations used, which can be used to enhance the description of the obtained results. Note that the first group of indicators corresponds to CIs, followed by entropy-based indicators (EIs), and finally CEIs.

In general terms, the classification accuracy scores of Table 2 are above 70%. Considering groups by indicator type (columns), the CIs group has the lowest values of accuracy for the classification, from 73% to 78%. The results improved by the next group, entropy-based indicators (EIs), where all scores are above 90%. The information given by the entropy-based indicators (EIs) group allows the RF classifier to have scores up to 99%, which is the classification of data at 5 rpm. The CIEs group has an overall higher accuracy score, which is a minimum of 90% and a maximum of 98%. Although the entropy-based indicators (EIs) group has the same lower accuracy percentage value as the CIEs group, and its best value is the highest from all accuracy score values calculated (99%), at 8 rpm, it is several percentage points lower than its similarity to the CIEs group. The $\sigma_{\mathrm{as}}$ also reveals the variability of the results of the same groups. From this data, the lowest $\sigma_{\mathrm{as}}$ value is in the group of CEI models at 8 rpm, and the highest in the CI group at 5 rpm. In general, the $\sigma_{\mathrm{as}}$ values from the CIs trained model group are higher than for the entropy-based indicators (EIs) and CIEs groups, with differences between 30% to 90%, in comparison to $\sigma_{\mathrm{as}}$ values from CIs at the same rpm.

A closer view of the results from Table 2 reveals that for the same indicator group, a better classification score is obtained for lower rotational speeds. For instance, the CIs group improves by 4 percentage points from 10 to 5 rpm. The same trend occurs in the EI and CEI groups by 9 and 8 percentage points, respectively. Table 2 provides a noteworthy difference among accuracy scores from the trained models of the CIs group, and the models from the EI and CEI groups. For example, at 10 rpm, the result from CIs is 17 percentage points lower than that from the entropy-based indicators (EIs) group, with a maximal of 22 percentage points with a difference at 5 rpm. Considering groups by rows (models for signals at same rpm), the accuracy scores at 10 rpm are the lowest for the three row group. The results for the 8 rpm group increase in value compared to the described group. In the case of the 5 rpm group, the classification score based on CIs is considerably lower than entropy-based indicators (EIs) and CIEs, with at least 20 percentage points below the corresponding values for the entropy-based indicators (EIs) and CIEs groups.

Table 2 gives a general view of the difference among classification according to several rotational speeds and types of used indicators. For a deeper analysis on each classification performance, Table 3 shows the classification accuracy score result for one particular trained model set, which is chosen randomly. To validate the trained model, a cross-validation approach of 5-fold was utilized. In addition, Table 4 shows the training time for all trained models.

**Figure 13:**
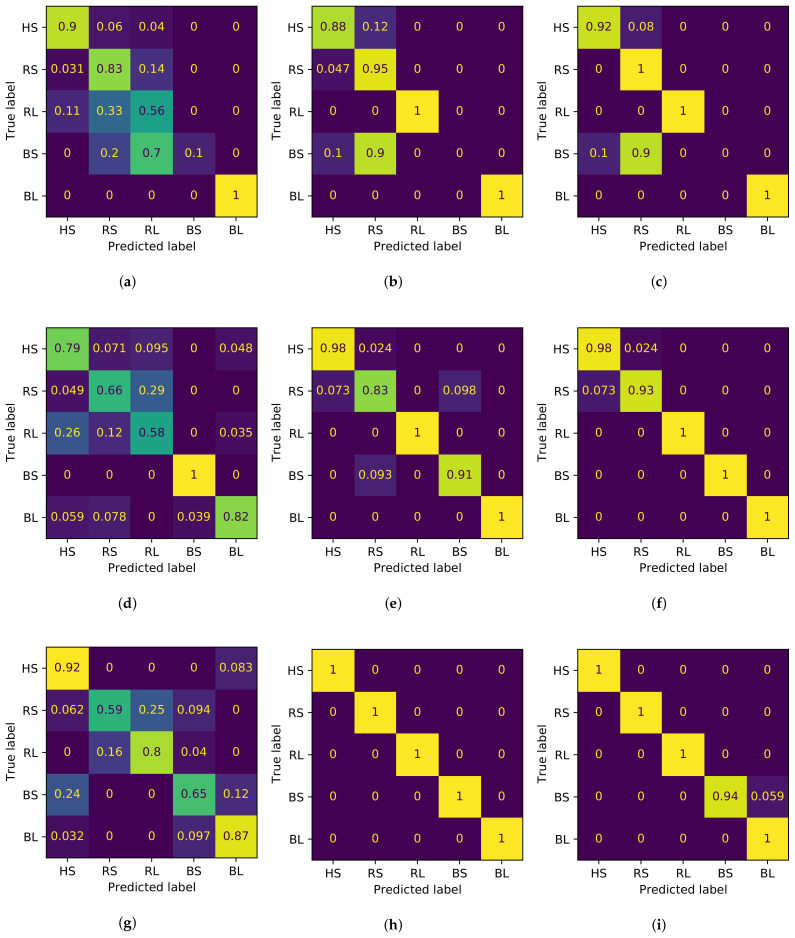
Confusion matrices for the diagnosis of low-speed bearings using random forest classifier, varying the indicator type and the rotational speed of the data. The results were normalized over the true labels. The first row shows the results at 10 rpm, the second row at 8 rpm, and the last row at 5 rpm. The first column shows the results for classic indicators (CIs), second column for entropy indicators (EIs), and the last column shows the classic and entropy indicators (CEIs). (**a**) Confusion matrix for CIs and data at 10 rpm. (**b**) Confusion matrix for EIs and data at 10 rpm. (**c**) Confusion matrix for CEIs with data at 10 rpm. (**d**) Confusion matrix for CIs and data at 8 rpm. (**e**) Confusion matrix for EIs and data at 8 rpm. (**f**) Confusion matrix for CEIs and data at 8 rpm. (**g**) Confusion matrix for CIs and data at 5 rpm. (**h**) Confusion matrix for EIs and data at 5 rpm. (**i**) Confusion matrix for CEIs and data at 5 rpm.

The results from Table 3 show that lower accuracy scores are related to the CIs column. The classification score is improved while the rotational speed decreases for all indicator types. It is worth mentioning the particular case of perfect classification at 5 rpm, by using entropy-based indicators (EIs). This result is followed by classification at 8 and 5 rpm, with the use of CIEs. Table 4 shows the training time for each model in Table 3. In general, the CIs group consumes the highest time from all groups at the same rotational speed, followed by CEIs and EIs. A detailed explanation of the behavior from the classifiers according to the type of indicator is presented in Figure 13, where the confusion matrices for all trained models from Table 3 are provided. It is important to note that the matrices are ordered in concordance with the values from Table 3, that is, columns represent indicator groups, whereas rows represent the different operating speeds of the bearing.

The confusion matrices for the classification at 10 rpm are shown in Figure 13a for CIs, Figure 13b for entropy-based indicators (EIs), and Figure 13c for CIEs. The confusion matrix in Figure 13a shows that the classification rates for the RL and BS scenarios are the lowest for all classification scores at 10 rpm. In particular, the RL is recognized as more than half of the data, while 70% of the BS data is classified as RL. The classification from Figure 13b has the same problem as the BS, where 90% of the data is confused as RS and the rest as HS. The HS and RS scenarios are recognized above 88% of the data, but some data are misclassified. Figure 13c shows that the same problem of classification of the BS data using the entropy-based indicators (EIs) occurs using the CIEs, where 90% of the data is classified as RS and 10% as HS. In contrast, RS is always well classified and the HS rate exceeds 90% of the data.

The confusion matrices for 8 rpm are shown in Figure 13d for CIs, Figure 13e for entropy-based indicators (EIs), and Figure 13f for CIEs. Although the accuracy score for this classification is 2 percentage points above the classification for CIs at 10 rpm, the confusion matrix shows the misclassification for all scenarios, except for BS. In contrast to the results in Figure 13a, RS and RL are the scenarios with the worst classification (66% and 58%, respectively). The previous situation is improved in Figure 13e, where entropy-based indicators (EIs) has a perfect score for RL and BS. While almost 10% of the BS is misclassified, the HS and RS classifications are improved by almost 20 percentage points. Furthermore, the BL classification is improved to the maximum value. Figure 13f denotes an improvement of the classification compared with the previously described confusion matrix (where RL, BS, and BL have perfect classification). In contrast, the scores of HS and RS are above 90%, and the misclassified values from each scenario are confused with the other aforementioned scenario, respectively.

The results for classification at 5 rpm are presented in Figure 13g–i. The trained model in Figure 13g has the lowest classification rate from all models at 5 rpm. In this figure, a critical case takes place, where a significant proportion of the data from all scenarios is misclassified. For the classification of RS, 59% of the values are correctly classified, which has the lowest accuracy rate compared with other scenarios. As approximately 40% of RS data is confused, the main problem is the variety of scenarios in which the misclassified data are categorized. In contrast, only data from RL are not confused as HS. The previous situation is improved using EIs and CIEs, as shown in Figure 13h,i, where the classification is generally congruent with the original data. The only exception is the classification of the BS in Figure 13i, where less than 6% of the data is misjudged as BL.

Considering the contrast of the scenarios, HS is correctly classified in all models. The worst accuracy score occurs in the CIs-based trained model at 8 rpm, with 79% of the rotations classified. Next, RS and RL are classified at a lower accuracy by the CIs group (with lower scores at 8 rpm) compared with the EIs and CIEs groups. Moreover, several RS rotations are confused as HS, but at 5 rpm, all rotations are perfectly scored by EIs and CIEs. For RL, the lower scores are also in the CIs group. However, BS and BL are classified with higher scores by the EIs and CIEs groups at all rotational speeds compared with the EIs group. The only exception is at 10 rpm, where 90% of the rotations are classified as RS and 10% as HS.

In general, the RF trained models correctly classified over 56% of the data from each bearing scenario at all rotational speeds. The models based on CIs classified HS rotations over 79% of the rotations as HS. Based on the scores obtained from classified damaged scenarios, the CIs group had some difficulty with respect to differentiating between them (in comparison to the entropy-based indicators (EIs) and CIEs groups), as some rotations from damaged scenarios are misclassified several times as rotations from a healthy bearing. The results show that the diagnosis by entropy-based indicators (EIs) of low-speed bearing is feasible. With accuracy scores of 98%, a healthy bearing can be accurately differentiated from a damaged bearing. As the majority of classification scores from damaged scenarios are higher than the scores obtained by the CIs group, it can be stated that the information extracted using entropy-based indicators (EIs) contributes to the classification of damaged bearings with greater accuracy than entropy-based indicators (EIs). In addition, the information extracted using EIs can contribute positively to CIs to distinguishing between healthy and damaged bearings. The evidence shows that CIEs correctly classified more than 93% of the times relative to the rotations of a signal from healthy bearings, which is higher than the CIs and EIs groups. Finally, as the rotational speed decreases, the improvement of the CIs group is nearly half of the improvement from the EIs and CIEs group. Hence, a possible correlation between the addition of EIs to the trained RF model is stated. This is a positive behavior for the rotational work speed of pitch bearings, which are as low as 1 rpm.

The classification of data with four EIs is compared with the same classification process using 16 CIs, and later the indicators are combined for a third classification campaign. The information from the data obtained by EIs enables a higher classification precision compared with the results obtained from the CIs classification. The available data, which includes several damage scenarios, show that the CIs classification is useful for determining the health of a bearing, but the declaration of the damage scenario is not as clear as with the EIs classification. The combination of both indicator types in the CIEs classification shows a general improvement in the results. Therefore, EIs can be considered as a complement for classification using CIs for better and more detailed diagnosis.

The results show the effectiveness of the entropy-based indicators for low-speed bearing diagnosis. However, several requirements are needed to implement these indicators in reality. Considering the technology readiness level of this research (level 4), the controlled laboratory environment, where no external perturbations are found, is still distant from reality. Therefore, the next step for future analysis is to validate the EIs in a relevant environment. From this perspective, the environment of pitch bearing is considerably noisier than a controlled laboratory environment. As said before, a wind turbine has several electromechanical parts, and each part contributes as an external perturbation to a vibration signal. As a result, these external perturbations can contribute negatively to the application of the EIs. Besides, the size and work regimen of the pitch bearings are needed to be considered for a final application. Another issue is the rotation of the pitch bearing, which is normally not a complete rotation. The main reason is the generation control system, which considers the random forces coming from the wind for the position of the blade against the wind. A subsequent study could bring the data set closer to reality, configuring the movement of the bearing clockwise and anticlockwise for further analysis.

The evolution of pitch bearings from a healthy state to a damaged one can last from months to years. Since the size of the data volume is unpractical for analysis, a meaningful time data acquisition per day is enough for monitoring systems. As the entropy-based indicators were calculated per second, independent of the speed, this can help its implementation in a monitoring system. One main conclusion from the results is the improvement of the information obtained from EIs at a lower rotational speed. This is particularly advantageous for its application. The focus of this work is the analysis of pitch bearings from wind turbines, but some other applications of low-speed bearings can be benefited from this method. For example, yaw bearings from wind turbines, slew bearings from cranes, or swing bearings from backhoe or excavator machinery.

## 6. Conclusions

In this paper, the use of entropy-based indicators for the classification of low-speed bearing vibration signals was proposed as an alternative to classic indicators used in the literature. To demonstrate the feasibility and benefits of entropy-based indicators (EIs), the classification of a set of bearings was proposed using vibration analysis. First, the signals from the data set were divided into time series. Then, entropy-based indicators were calculated, in addition to classic indicators used in the literature. RF models were trained based on previous results, to classify the time series. The data set employed had multiple damage scenarios at various low rotational speeds hence, the contrast among rotational speed, damage scenario types (including damage position and size), and indicators is stated.

The results showed that EIs and its use for the classification of low-speed bearing vibration signals was feasible as useful information from the signals could be obtained for diagnosis. In addition, the findings indicated that 4 EIs can be classified with an accuracy score of up to 22 percentage points above the scores obtained by 16 CIs for 5, 8, and 10 rpm. The research also demonstrated that for lower rotational speeds, the data classification score obtained using EIs could be more than 20 percentage points higher than the CIs. Finally, the results show that the use of EIs in conjunction with CIs could classify a dataset with higher accuracy compared to the classification with the indicators separately. This shows the ability of EIs to differentiate among damaged scenarios, which is an improvement in comparison to the CIs classification, which is beneficial for the diagnosis of low-speed bearings.

As the dataset used for this study has several controlled laboratory conditions, multiple challenges must be overcome before the application of EIs in systems in operational environments. For example, the forces applied to the bearing in this study were always continuous as well as the rotational speed. Thus, one of the following steps for the study consists of the design and execution of tests with variable applied forces. Moreover, because pitch bearings rotate by several degrees each time, reproducing such cyclical movement could increase the proximity to real operating conditions, in contrast to the constant rotational speed used in this study. The addition of variable rotational speeds can also help to conceptualize the boundaries of EIs. Finally, the suitability of the EIs for scenarios with rotational speeds lower than 5 rpm could be analyzed.

The damaged bearings used in this study differ from each other based on the location or size of the damage. A further study could combine the location of several damages to include scenarios where a combination of ball and raceway (or even cage) can occur. An increase in the complexity of the damage by introducing irregularities in the surfaces or testing naturally degraded bearings could also prove useful not only to improve the analysis of the current state by the use of EIs, but to also state the basis for prognosis based on EIs.

## Figures and Tables

**Table 1:** Number of rotations for healthy scenario (HS), small raceway shaft washer damage scenario (RS), large raceway shaft washer damage scenario (RL), small ball bearing damage scenario (BS), and large ball bearing damage scenario (BL) at 5, 8, and 10 revolutions per minute (rpm).

	HS	RS	RL	BS	BL
10 rpm	191	195	195	40	80
8 rpm	156	156	156	156	156
5 rpm	97	97	97	97	97

**Table 2:** Average ${\bar{x}}_{\mathrm{as}}$ and standard deviation $\sigma_{\mathrm{as}}$ of the accuracy classification score from 1000 trained models of random forest classifier by the use of classic indicators (CIs), entropy indicators (EIs), and classic and entropy indicator (CEIs) for signals at 5, 8, and 10 rpm.

	CIs		EIs		CEIs	
	${\bar{x}}_{\mathrm{as}}$	$\sigma_{\mathrm{as}}$	${\bar{x}}_{\mathrm{as}}$	$\sigma_{\mathrm{as}}$	${\bar{x}}_{\mathrm{as}}$	$\sigma_{\mathrm{as}}$
10 rpm	73%	0.027285	90%	0.017744	90%	0.018584
8 rpm	78%	0.025358	94%	0.013072	97%	0.010761
5 rpm	77%	0.033270	99%	0.003561	98%	0.014807

**Table 3:** Accuracy score of random forest classifier by the use of classic indicators (CIs), entropy indicators (EIs), and classic and entropy indicator (CEIs) for signals at 5, 8, and 10 rpm.

	CIs	EIs	CEIs
10 rpm	74%	90%	93%
8 rpm	76%	94%	98%
5 rpm	75%	100%	98%

**Table 4:** Training time (in seconds) of random forest classifier by the use of classic indicators (CIs), entropy indicators (EIs), and classic and entropy indicator (CEIs) for signals at 5, 8, and 10 rpm.

	CIs	EIs	CEIs
10 rpm	1.7091	1.5192	1.6991
8 rpm	1.7624	1.5625	1.7391
5 rpm	1.5992	1.4790	1.5792

## Data Availability

Data sharing is not applicable to this article.

## Abbreviations

The following abbreviations are used in this manuscript:

AppEn	approximate entropy
BL	large ball bearing damage scenario
BS	small ball bearing damage scenario
CBM	condition-based maintenance
CEI	classic and entropy indicators
CF	crest factor
CI	classic indicator
CM	condition monitoring
DisEn	dispersion entropy
DT	decision tree
EI	entropy indicator
EMD	empirical mode decomposition
EU	European Union
FC	frequency center
HS	healthy scenario
IF	impulse factor
IMF	intrinsic mode function
MF	margin factor
NCDF	normal cumulative distribution function
O&M	operating and maintenance
PerEn	permutation entropy
PSD	power spectral density
RF	random forest
RL	large raceway shaft washer damage scenario
RMS	root mean square
RMSF	root mean square frequency
rpm	revolutions per minute
RS	small raceway shaft washer damage scenario
RVF	root variance frequency
SepEn	spectral entropy of the permutation entropy signal
SF	shape factor
SpeEn	spectral entropy
SvdEn	singular value decomposition entropy
